# Minimum Volume Standards: An Incentive To Perform More Radical Cystectomies?

**DOI:** 10.1016/j.euros.2023.02.015

**Published:** 2023-03-31

**Authors:** Siberyn T. Nuijens, Lisa M.C. van Hoogstraten, Richard P. Meijer, Lambertus A. Kiemeney, Katja K.H. Aben, J. Alfred Witjes

**Affiliations:** a Department of Urology, Radboud University Medical Centre, Nijmegen, The Netherlands; b Netherlands Comprehensive Cancer Organisation, Utrecht, The Netherlands; c Radboud Institute for Health Sciences, Radboud University Medical Centre, Nijmegen, The Netherlands; d Department of Oncological Urology, University Medical Centre Utrecht, Utrecht, The Netherlands

**Keywords:** Bladder cancer, Minimum volume standards, Radical cystectomy

## Abstract

**Background:**

Minimum volume standards (MVS) for hospitals and/or surgeons remain a subject of debate. Opponents of MVS emphasize the possible negative effects of centralization, such as an unwanted incentive to perform surgery.

**Objective:**

To evaluate whether the introduction of MVS for radical cystectomy (RC) in the Netherlands resulted in more RCs outside guideline-recommended indications.

**Design, setting, and participants:**

All RCs performed for bladder cancer in the Netherlands between January 1, 2006 and December 31, 2017 were identified in the Netherlands Cancer Registry. During this period, two MVS were sequentially implemented for RC. RCs in intermediate-volume hospitals (hospitals that approximated the MVS) were compared with RCs in high-volume hospitals (hospitals exceeding the MVS by ≥5 RCs/yr) in a period before and a period after implementation of each of the two MVS.

**Outcomes measurements and statistical analysis:**

Descriptive analyses were performed to evaluate whether hospitals performed more RCs outside the recommended indication (cT2–4a N0 M0) and whether an increase in the number of RCs towards the end of the year could be observed.

**Results and limitations:**

After MVS implementation, no clear shift towards disease stages outside the recommended indication for RC was observed in comparison to the period before the MVS. Results for high-volume and intermediate-volume hospitals were similar. In addition, no increase in RCs towards the end of the year was evident.

**Conclusions:**

We did not find evidence indicating an unwanted incentive to perform more RCs as a result of MVS in the Netherlands. Our results further strengthen the case for MVS implementation.

**Patient summary:**

We evaluated whether criteria for the minimum number of radical cystectomies (surgical removal of the bladder) that hospitals have to perform caused urologists to perform more of these operations than necessary in order to meet the minimum level. We found no evidence that minimum criteria led to such an unwanted incentive.

## Take Home Message

Minimum volume standards for radical cystectomy do not seem to create an unwanted incentive to perform more surgeries.

## 1 Introduction

Minimum volume standards (MVS) for hospitals and surgeons have been a subject of discussion since 1979, when Luft et al [1] hypothesized that there is a relationship between higher surgical volume and lower postoperative mortality. The idea behind this relationship is the “practice makes perfect” principle, whereby patients in high-volume hospitals (HVHs) would benefit from a higher degree of surgical experience than patients in low-volume hospitals (LVHs). Surgical experience applies not only to the surgical team performing the procedure but also to the ward staff, facilities, and infrastructure involved in postoperative care [2], [3].

This volume-outcome relationship has been extensively studied across multiple surgical fields [4], [5], [6]. In 2002, Birkmeyer et al [4] investigated 14 types of cardiovascular and cancer surgeries in a cohort of 2.5 million procedures and found lower mortality for higher hospital volume for all 14 procedure types. Similarly, multiple systematic reviews found lower mortality or complication rates in hospitals with higher volume for different types of surgery [7], [8], [9], [10], [11].

Regarding oncological urology, several studies have shown a clear volume-outcome relationship for radical cystectomy (RC) and the European Association of Urology (EAU) recommends that hospitals perform at least ten and preferably more than 20 RCs annually [2], [12]. In the past 20 yr, multiple countries have introduced MVS for different types of surgical procedures, including RC [13]. In the Netherlands, an MVS of 10 RCs for bladder cancer per hospital per year, using 3-yr averages, was introduced by the Dutch Urology Association in 2010. In 2015 this MVS was raised to 20 RCs annually per hospital.

MVS and their effects on the centralization of health care remain controversial. MVS opponents emphasize their possible negative consequences. For instance, centralization leads to fewer training opportunities for junior staff. Patients and family members could experience a greater travel burden not only for the procedure itself but also for preoperative and postoperative appointments. For patients in rural areas, this can ultimately lead to reduced access to care. Finally, MVS implementation can result in an unwanted incentive to perform surgery. For example, low-grade and/or low-stage bladder cancer normally does not require radical surgical treatment, but urologists might still be inclined to perform RC in such patients to meet the MVS [14], [15], [16], [17]. In a qualitative interview study from 2018, Dutch surgeons reported that this undesired strategic behavior sometimes occurs [14]. Similarly, Hlatky [15], Schwartz et al [16], and Stanak and Strohmaier [7] all debated the possibility of this “perverse incentive” as a result of MVS implementation. The objective for the current study was to examine whether MVS lead to an unwanted incentive to perform more RCs. To this end, we analyzed data from all hospitals that performed RCs in the Netherlands between 2006 and 2017. We hypothesized that if an MVS results in an unwanted incentive for hospitals to perform more RCs, an increase in the number of RCs outside the recommended indication (cT2–4a N0 M0 and high-risk non–muscle-invasive bladder cancer), such as for non–high-risk stage cT1 and/or advanced stage cT4 Nx Mx disease, might be observed, which we later refer to as a *stage-shift hypothesis*. In addition, an increase in the number of RCs performed in the final quarter of the year (later referred to as an *end-of-year-sprint hypothesis*) might be observed. These effects might be expected in hospitals for which the annual number of RCs did not meet but approximated the MVS and in the first 3 yr after MVS implementation, since MVS adherence is determined using 3-yr averages.

## 2 Patients and methods

### 2.1 Cohort and data

For this nationwide historical cohort study, data from the Netherlands Cancer Registry (NCR) were used. The NCR is a nationwide population-based registry started in 1989 that covers the entire Dutch population of approximately 17 million inhabitants. In the NCR, identification of newly diagnosed malignancies is mainly based on notification from the nationwide network and registry of histopathology and cytopathology in the Netherlands (PALGA). The NCR contains data on patient and tumor characteristics and disease stage. Topography and morphology are coded according to the International Classification of Diseases for Oncology (ICD-O-3) [18]. Tumors were staged according to the Union Internationale Contre le Cancer TNM classification applicable at the time of diagnosis [19]. In addition, the initial treatment for each first noninvasive bladder cancer and first invasive or muscle-invasive bladder cancer are recorded in the NCR. Regarding RC specifically, from 2012 onwards all RCs were recorded, including RCs performed in cases of progression from T1 to muscle-invasive disease.

All RCs for bladder cancer, regardless of stage and histology, performed in a Dutch hospital between January 1, 2006 and December 31, 2017 were selected from the NCR. Data on patient (age, sex), tumor (TNM stage), and treatment (neoadjuvant treatment, RC date, and hospital) characteristics were retrieved. The study was approved by the Privacy Review Board of the NCR, and did not require approval from an ethics committee.

### 2.2 Time period and hospital volume

To evaluate the effect of the MVS of 10 RCs/hospital/year in 2010 and of 20 RCs/hospital/year in 2015, two different time periods were defined. The first period covers 2010–2012, which includes the 3 yr after introduction of the first MVS (period 1); the second period covers 2015–2017, the 3 yr after the new MVS (period 2). For each period, hospitals were divided in two groups according to the total number of RCs performed in the year before MVS implementation: (1) intermediate-volume hospitals (IVHs), which did not meet but approximated the MVS; and (2) HVHs, which exceeded the MVS.

For analyses for period 1, IVHs are defined as hospitals with a volume of 6–10 RCs in the year preceding the newly introduced MVS (2009). HVHs were defined as hospitals that performed ≥15 RCs in 2009. For analyses for period 2, a hospital was defined as an IVH if it performed 16–20 RCs in the year preceding the MVS (2014) and as a HVH if it performed ≥25 RCs in 2014. For both periods, hospitals that stopped performing RCs in the first 3 yr after implementation of the MVS were excluded (*n* = 6 for period 1 and *n* = 4 for period 2).

### 2.3 Statistical analysis

Descriptive statistics were calculated to describe the IVH and HVH cohorts from period 1 and period 2. To test the stage-shift hypothesis, the distribution of clinical disease stage was compared between the IVH and HVH settings for all patients who underwent RC. To evaluate the effect of the MVS, disease stage distribution for patients undergoing RC in IVH and HVH settings after MVS implementation was compared with the distribution in a period before the MVS. The reference period was 2006–2008 for period 1 and 2011–2013 for period 2. To test the end-of year-sprint hypothesis, RCs were grouped by quarter according to the date of surgery. Since hospitals report their volumes on a yearly basis, it was assumed that, given the hypothesis, an increase in the number of RCs in the final quarter of the year would be expected. Therefore, the proportion of RCs in the final quarter of the year was compared between the IVH and HVH settings, and to the reference years for period 1 and period 2.

All analyses were performed in SAS v9.4 (SAS Institute, Cary, NC, USA).

## 3 Results

### 3.1 Patient characteristics

In total, 9608 patients with bladder cancer who underwent RC in the Netherlands between 2006 and 2017 were selected from the NCR. The RCs were performed in 95 different hospitals.

Table 1 presents the patient and tumor characteristics for those treated with RC in the IVH and HVH settings during period 1 (*n* = 1410). Among these patients, 15% had non–muscle-invasive bladder cancer (NMIBC), 52% had cT2 stage muscle-invasive bladder cancer (MIBC), and 28% had cT3+ stage MIBC. There was no difference in sex distribution between the groups, while the median age was 68 yr in the IVH cohort and 67 yr in the HVH cohort (*p* = 0.003). More patients received neoadjuvant treatment (radiotherapy, chemotherapy, or both) in the HVH setting than in the IVH setting (17% vs 8%; *p* < 0.001).

**Table 1:** Patient and tumor characteristics for all patients treated with RC in IVH and HVH settings in the Netherlands between 2010 and 2012

	IVH setting	HVH setting	*p* value
	(23 hospitals)	(11 hospitals)	
Patients (*n*)	708	702	
Male, *n* (%)	520 (73)	515 (73)	>0.9 a
Median age at diagnosis, yr (interquartile range)	68 (62–74)	67 (60–73)	0.003 b
Age group at diagnosis, *n* (%)			0.020 a
<60 yr	117 (17)	158 (23)	
60–70 yr	286 (40)	273 (39)	
70–80 yr	259 (37)	220 (31)	
≥80 yr	46 (6.5)	51 (7.3)	
Clinical disease stage, *n* (%)			0.064 a
Ta(i)	6 (0.8)	10 (1.4)	
Tis	27 (3.8)	28 (4.0)	
T1(i) N0 M0	83 (12)	62 (8.8)	
T2 N0 M0	390 (55)	349 (50)	
T3 N0 M0	69 (9.7)	74 (11)	
T4a N0 M0	24 (3.4)	32 (4.6)	
T4b and/or cN+ and/or cM+	83 (11)	114 (16)	
Unknown	26 (3.7)	33 (4.7)	
Neoadjuvant treatment, *n* (%)			<0.001 a
None	652 (92)	581 (83)	
Neoadjuvant chemotherapy	53 (7.5)	116 (17)	
Neoadjuvant radiotherapy	3 (0.4)	3 (0.4)	
Both	0 (0.0)	2 (0.3)	
Year of RC, *n* (%)			0.4 a
2010	204 (29)	187 (27)	
2011	251 (37)	242 (35)	
2012	253 (36)	273 (39)	

HVH = high-volume hospital (≥15 RCs/yr); IVH = intermediate-volume hospital (6–10 RCs/yr); RC = radical cystectomy.

a χ^2^ test.

b Analysis-of-variance F test.

Table 2 lists the patient and tumor characteristics for those treated with RC in the IVH and HVH settings in period 2 (*n* = 1356) are presented. Among these patients, 18% had NMIBC, 46% had cT2 stage MIBC, and 32% had cT3+ stage MIBC. During period 2, the sex distribution was similar between the two settings; the median age was 70 yr in the IVH cohort and 68 yr in the HVH cohort (*p* < 0.001). Some 20% of patients treated in HVHs received neoadjuvant treatment versus 16% of patients in IVHs (*p* = 0.2).

**Table 2:** Patient and tumor characteristics for all patients treated with RC in IVH and HVH settings in the Netherlands between 2015 and 2017

	IVH setting	HVH setting	*p* value
	(6 hospitals)	(10 hospitals)	
Patients **(***n*)	314	1042	
Male, *n* (%)	228 (73%)	743 (71%)	0.7 a
Median age at diagnosis, yr (interquartile range)	70.0 (64–75)	68.0 (61–74)	<0.001 b
Age group at diagnosis, *n* (%)			0.022 a
<60 yr	45 (14)	210 (20)	
60–70 yr	107 (34)	389 (37)	
70–80 yr	136 (43)	367 (35)	
≥80 yr	26 (8.3)	76 (7.3)	
Clinical disease stage, *n* (%)			0.035 a
Ta(i)	12 (3.8)	33 (3.2)	
Tis	5 (1.6)	42 (4.0)	
T1(i) N0 M0	39 (12)	112 (11)	
T2 N0 M0	167 (53)	461 (44)	
T3 N0 M0	36 (12)	170 (16)	
T4a N0 M0	13 (4.1)	46 (4.4)	
T4b and/or cN+ and/or cM+	32 (10)	136 (13)	
Unknown	10 (3.2)	42 (4.0)	
Neoadjuvant treatment, *n* (%)			0.2 a
None	263 (84)	832 (80)	
Neoadjuvant chemotherapy	51 (16)	201 (19)	
Neoadjuvant radiotherapy	0 (0)	5 (0.5)	
Both	0 (0)	4 (0.4)	
Year of radical cystectomy, *n* (%)			0.8 a
2015	101 (32)	317 (30)	
2016	102 (33)	359 (35)	
2017	111 (36)	366 (35)	

HVH = high-volume hospital (≥25 RCs/yr); IVH = intermediate-volume hospital (16–20 RCs/yr); RC = radical cystectomy.

a χ^2^ test.

b Analysis-of-variance F test.

### 3.2 Stage-shift hypothesis

**Fig. 1:**
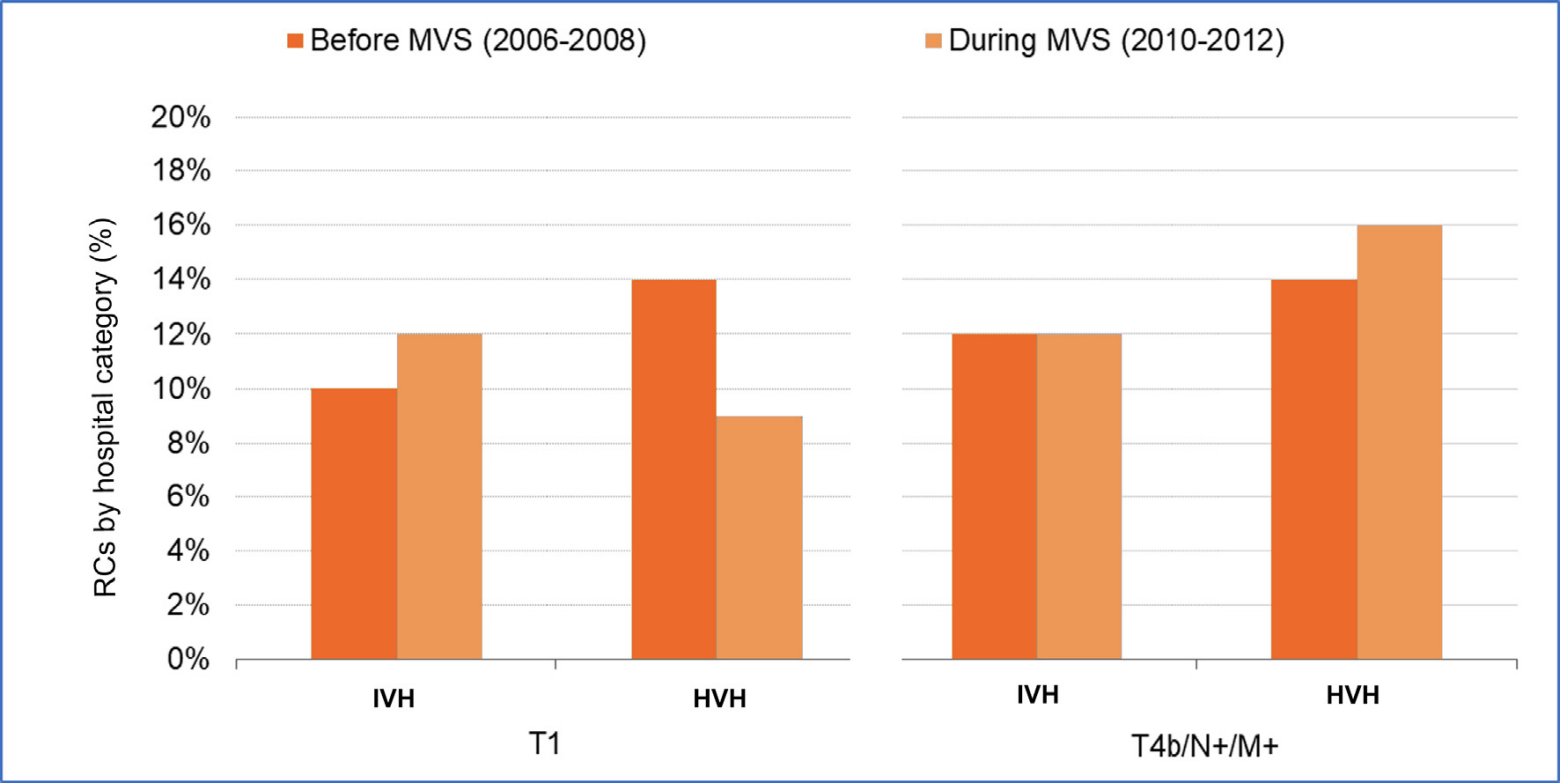
Proportion of radical cystectomies (RCs) for T1 and T4b disease stages in intermediate-volume hospitals (IVH; 6–10 RCs/yr) and high-volume hospitals (HVH; ≥15 RCs/yr) during 2006–2008 (before the introduction of the first minimum volume standard [MVS]) and 2010–2012 (after the introduction of the first MVS). There was no significant increase in the proportion of RCs for T1 and/or T4b/N+/M+ tumors after implementation of the first MVS (2010).

Figure 1 shows the proportion of patients with cT1 and cT4b disease in the RC cohort for IVHs (23 hospitals) and HVHs (11 hospitals) in period 1 (2010–2012) and the relevant reference period (2006–2008). The distribution of other disease stages during these periods is shown in Supplementary Figure 1. The proportion of RCs performed for cT1 disease in IVHs was 10% before MVS implementation, which increased by 1.9% (95% confidence interval [CI] −1.7% to 5.4%) to 12% after MVS implementation. The proportion of RCs performed for cT1 disease in HVHs decreased by 5.6% (95% CI −9.3% to −1.9%) from 14% to 9% after MVS implementation. Regarding more advanced disease stages, 12% of patients treated with RC in IVHs had cT4 and/or N+ and/or M+ stage (cT4/N+/M+). This percentage did not change after MVS implementation (95% CI −3.7% to 3.7%). For HVHs the percentage of cT4/N+/M+ cases increased by 2.4% (95% CI −1.6% to 6.4%) from 14% to 16% after MVS implementation.

**Fig. 2:**
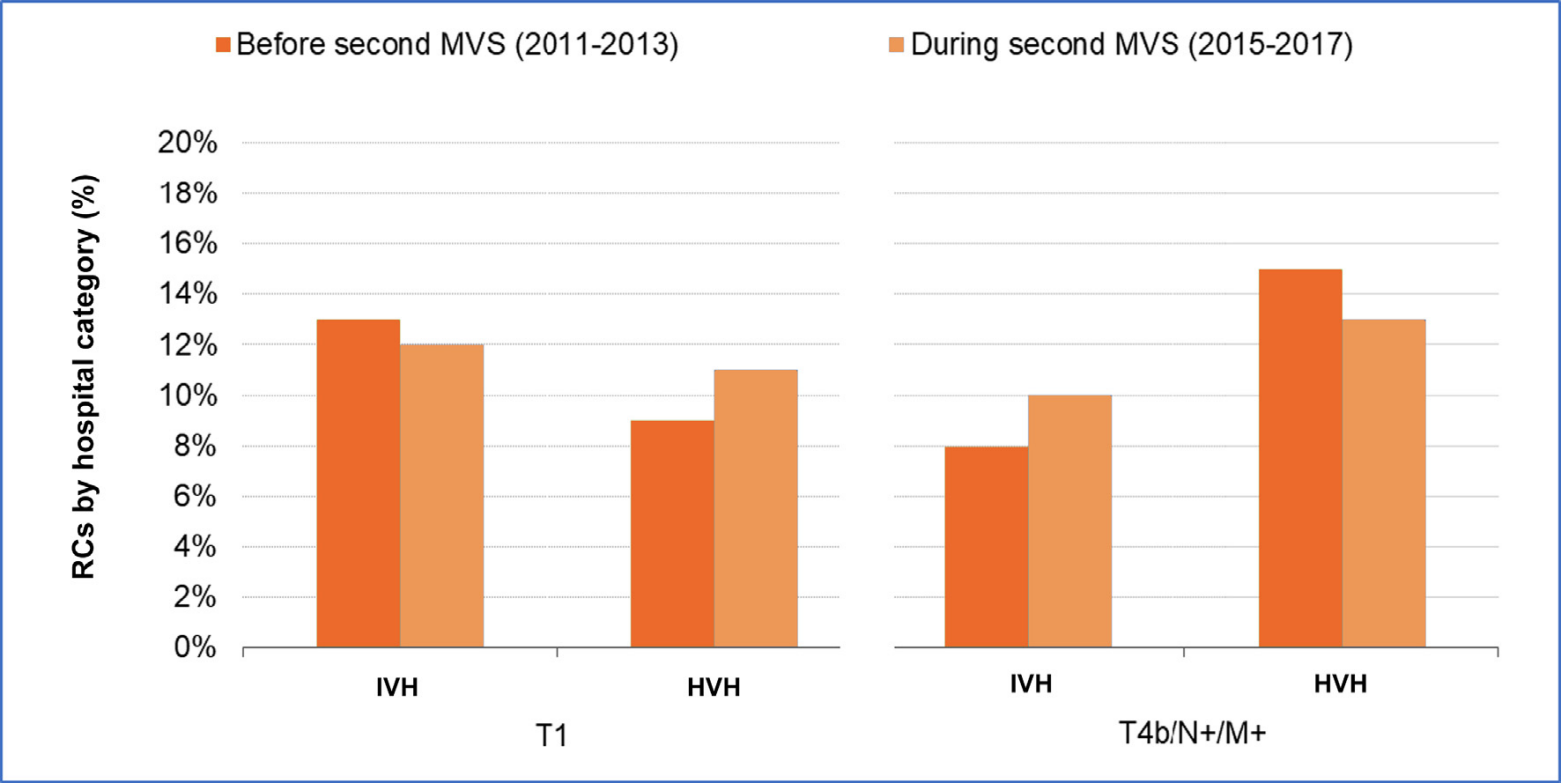
Proportion of T1 and T4b cases undergoing radical cystectomy (RC) in intermediate-volume hospitals (IVH; 16–20 RCs/yr) and high-volume hospitals (HVH; ≥25 RCs/yr) during 2011–2013 (before introduction of the second minimum volume standard [MVS]) and 2015–2017 (after introduction of the second MVS). There was no significant increase in the proportion of RCs for T1 and/or T4b/N+/M+ tumors after implementation of the second MVS (2015).

Regarding the second MVS, Figure 2 shows the proportion of T1 and T4b cases in the RC cohort for IVHs (6 hospitals) and HVHs (10 hospitals) in period 2. The reference period for the second MVS was 2011–2013. The distribution of other disease stages for the RC cohort during this period is shown in Supplementary Figure 2. During the reference period, 13% of tumors treated with RC in IVHs were stage cT1, which decreased by 0.3% (95% CI −5.7% to 5.1%) to 12% in period 2. In HVHs there was a corresponding increase of 1.6% (95% CI −1.6% to 3.8%) from 9% to 11%. cT4/N+/M+ stages accounted for 8% of all tumors treated with RC in IVHs in the reference period, which increased by 2.0% (95% CI −2.8% to 6.7%) to 10% in period 2. For HVHs this proportion decreased from 15% by 2.1% (95% CI −4.6% to 1.9%) to 13% after implementation of the second MVS. Overall, there was no clear stage shift following implementation of the two MVS.

### 3.3 End-of-year-sprint hypothesis

**Fig. 3:**
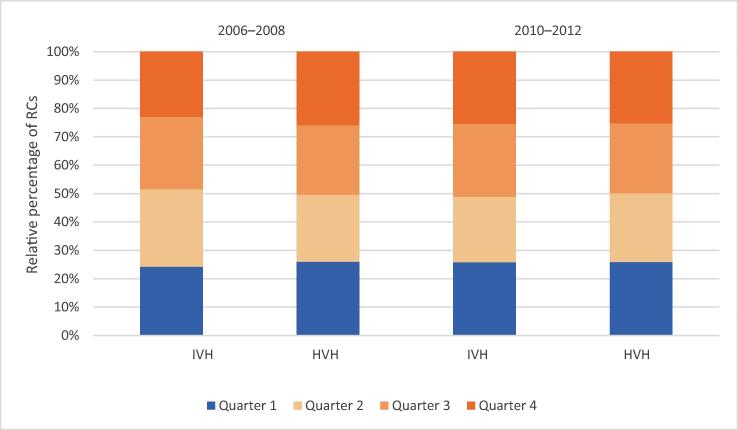
Distribution of radical cystectomies (RCs) by quarter in intermediate-volume hospitals (HVH; 6–10 RCs/yr) and high-volume hospitals (HVH; ≥15 RCs/yr) in period 1 (2010–2012) and the reference period (2006–2008).

**Fig. 4:**
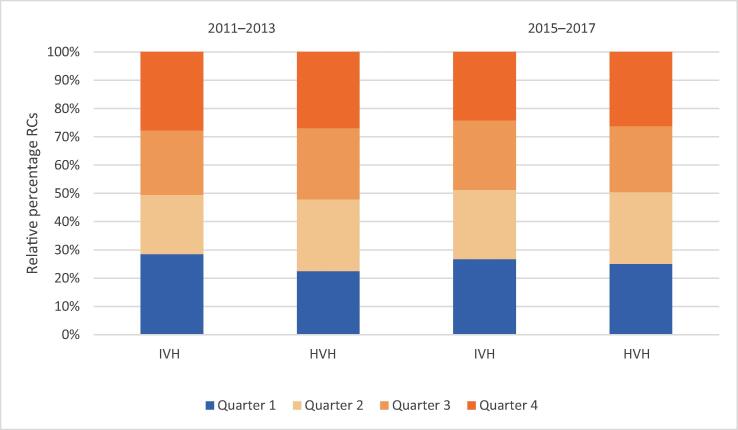
Distribution of radical cystectomies (RCs) by quarter in intermediate-volume hospitals (IVH) and high volume hospitals (HVH) in period 2 (2015–2017) and the reference period (2011–2013) [2].

Regarding the end-of-year-sprint hypothesis, Fig. 3, Fig. 4 show the distribution of RCs by quarter in period 1 and period 2, respectively, with comparison to the relevant reference period. In both periods, RCs were evenly distributed over the four quarters, similar to the reference periods, and there is no indication of an increase in the number of RCs towards the end of the year.

## 4 Discussion

In this nationwide cohort study we did not find any convincing evidence that MVS gave an unwanted incentive to perform more RCs in patients with bladder cancer in the Netherlands. Notably, this was also not found in hospitals that approximated the MVS, during the first years after its implementation. There were no indications of a shift in disease stage or of an increase in RCs in the final quarter of the year after MVS implementation. The proportion of RCs for cT1 and advanced disease stages remained stable after implementation of the MVS. In the Netherlands, RC is indicated in patients with cT2–4a N0/Nx M0 disease, in accordance to the EAU guidelines for MIBC [20]. For cT1 tumors, in contrast to some other countries, RC is only indicated in bacillus Calmette-Guérin–unresponsive patients and is generally not performed for treatment-naïve high-grade cT1 tumors. Therefore, a sudden increase in the number of RCs for cT1 tumors after implementation of a new MVS could have been an indication of an MVS-induced incentive to perform more RC procedures.

In addition, there was no increase in the proportion of patients with cT1 or advanced-stage bladder cancer treated with RC in IVHs compared to HVHs. In fact, HVHs had a slightly higher proportion of cT4/N+/M+ tumors treated with RC in all periods evaluated. Similar results were observed in a Dutch study on hospital volume for esophageal resections: Wouters et al [21] found that stage IV disease accounted for 17% of resections in HVHs versus 6% in LVHs. This could be explained by the fact that HVHs are often large hospitals with more expertise in this specific type of surgery. Patients with advanced disease stages are more likely to be referred to more experienced hospitals and those hospitals might be more inclined to perform surgery on advanced tumors. Furthermore, our results do not support the end-of-year-sprint hypothesis. All RCs were divided equally among the four quarters and there was no evidence of an increase in the number of RCs in the final quarter of the year. Furthermore, the median and mean ages for patients undergoing RC remained fairly stable in all quarters; we found no indication of a push to perform RC in older patients towards the end of the year. In order to prevent possible misclassification, we also performed an analysis using hospital volume based on the average number of RCs for the 3 yr before implementation of the MVS (The Dutch Urology Association also uses 3-yr averages in their definition of hospital volume). These analyses yielded similar results. Our data show that in 2006, 11% (67/593) of patients were treated with RCs in hospitals with a minimum volume of 20 RCs per year, which increased to 90% (844/942) in 2017. We can thus conclude that MVS did have the intended effect on centralization of RC in the Netherlands. A recent study by Richters et al [22], who used data from the NCR and included all RCs performed between 2008 and 2018, showed that the number of hospitals in the Netherlands performing RCs decreased from 86 in 2008 to 36 in 2018 as a result of MVS implementation.

To the best of our knowledge, this is the first nationwide study to examine whether MVS implementation represents an unwanted incentive to perform more surgeries in addition to the intended effect of centralization. Data for all hospitals that performed RCs between 2006 and 2017, as well as all tumor and patient data for the RCs, were available through the NCR, so our study results represent the effect of MVS implementation on a nationwide basis. Lastly, implementation of two different MVS in the Netherlands was included. This provides insights into the effects of nationwide introduction of a new volume standard, as well as the consequences of setting an even stricter MVS.

The findings from our study should be viewed in light of some limitations. First, the IVH cohort in period 2 (2015–2017) included only six hospitals and 314 patients. Because of these small numbers, we cannot draw strong conclusions from the data. In addition, we aggregated the data on stage distribution for the first 3 yr after MVS implementation, as the numbers were too small to investigate on a year-to-year basis.

Second, we categorized hospitals as IVHs and HVHs using volume as a dichotomous variable. If possible, the use of nonlinear splines to build a model with volume as a continuous variable is preferred over dichotomization. However, building a sufficiently flexible model given the relatively low number of hospitals included in the current study is potentially problematic. Hence, we opted for a simpler modeling strategy. Future studies in other countries that include more hospitals should consider use of more flexible modeling strategies with volume as a continuous variable. Third, up to 2012, only RCs that were part of the initial treatment for the first noninvasive or first (muscle-)invasive bladder cancer were recorded in the NCR. Therefore, the number of RCs performed for MIBC after an earlier diagnosis of T1 disease before 2012 will be slightly higher than reported. We do not expect this to have affected our results, since our study focused specifically on a stage shift in RCs for cT1 and/or cT4b bladder cancer. Fourth, we did not investigate the effect of MVS on oncological outcomes. However, this was previously studied by Richters et al [22], who found that 30-d and 90-d mortality slightly increased with hospital volumes up to 25 RCs/yr and decreased thereafter, without an indication of a plateau beyond a certain hospital volume. Finally, although there are no clear effects of MVS on a national scale, there may be individual surgeons who relaxed the indication for RC. Unfortunately, we could not evaluate surgeon volume since this information is not available through the NCR because of privacy legislation.

Notwithstanding its limitations, the current study contributes useful results to the ongoing debate regarding MVS criteria. While there is convincing evidence of positive effects on the mortality and morbidity associated with complex surgery [4], [5], [6], there are opponents of centralization who emphasize the possible negative effects of MVS. These include greater travel times, reduced access to care, limitations on teaching opportunities for junior staff, and the possibility of an unwanted incentive to perform more surgeries [14], [15], [23], [24], [25]. Some studies have addressed these problems. For instance, Hentschker and Mennicken [26] reported that centralization of care in Germany for patients with an abdominal aortic aneurysm or hip fracture improved outcomes without compromising overall access to care with regard to travel time. In any case, for a relatively small country such as the Netherlands, travel time is not expected to be a major limiting factor for centralization. A study by Xia et al [27] on associations between travel distance, hospital volume, and outcomes for patients undergoing RC concluded that the benefits of undergoing RC at a HVH outweigh the potential disadvantages of a longer travel distance. However, studies addressing the possibility of an MVS-induced unwanted incentive to perform more surgeries were lacking, which was the motivation for the present work.

## 5 Conclusions

In conclusion, we found no evidence of an unwanted incentive to increase the indication for radical surgery because of MSV introduction for RC. This result adds to the growing body of literature that favors MVS implementation.

  ***Author contributions***: J. Alfred Witjes had full access to all the data in the study and takes responsibility for the integrity of the data and the accuracy of the data analysis.

  *Study concept and design*: Nuijens, van Hoogstraten, Meijer, Kiemeney, Aben, Witjes.

*Acquisition of data*: Hoogstraten, Nuijens.

*Analysis and interpretation of data*: Nuijens, van Hoogstraten, Meijer, Kiemeney, Aben, Witjes.

*Drafting of the manuscript*: Nuijens.

*Critical revision of the manuscript for important intellectual content*: van Hoogstraten, Meijer, Kiemeney, Aben, Witjes.

*Statistical analysis*: van Hoogstraten.

*Obtaining funding*: None.

*Administrative, technical, or material support*: van Hoogstraten, Aben.

*Supervision*: Aben, Witjes.

*Other*: None.

  ***Financial disclosures*:** J. Alfred Witjes certifies that all conflicts of interest, including specific financial interests and relationships and affiliations relevant to the subject matter or materials discussed in the manuscript (eg, employment/affiliation, grants or funding, consultancies, honoraria, stock ownership or options, expert testimony, royalties, or patents filed, received, or pending), are the following: None.

  ***Funding/Support and role of the sponsor***: None.
