# Supplementary material for: Minimum Volume Standards: An Incentive To Perform More Radical Cystectomies?
Source: Eur Urol Open Sci. 2023 Mar 31;51:47–54. doi: 10.1016/j.euros.2023.02.015 (PMC10175736; doi:10.1016/j.euros.2023.02.015)
Supplement: Supplementary data 1 [file mmc1.docx]

Supplementary figure 1: Distribution of other disease stages for intermediate-volume (6-10 RCs/year) and high-volume (≥15RCs/year) hospitals during 2006-2008 (before the introduction of the first minimum volume standard) and 2010-2012 (after the introduction of the first minimum volume standard).


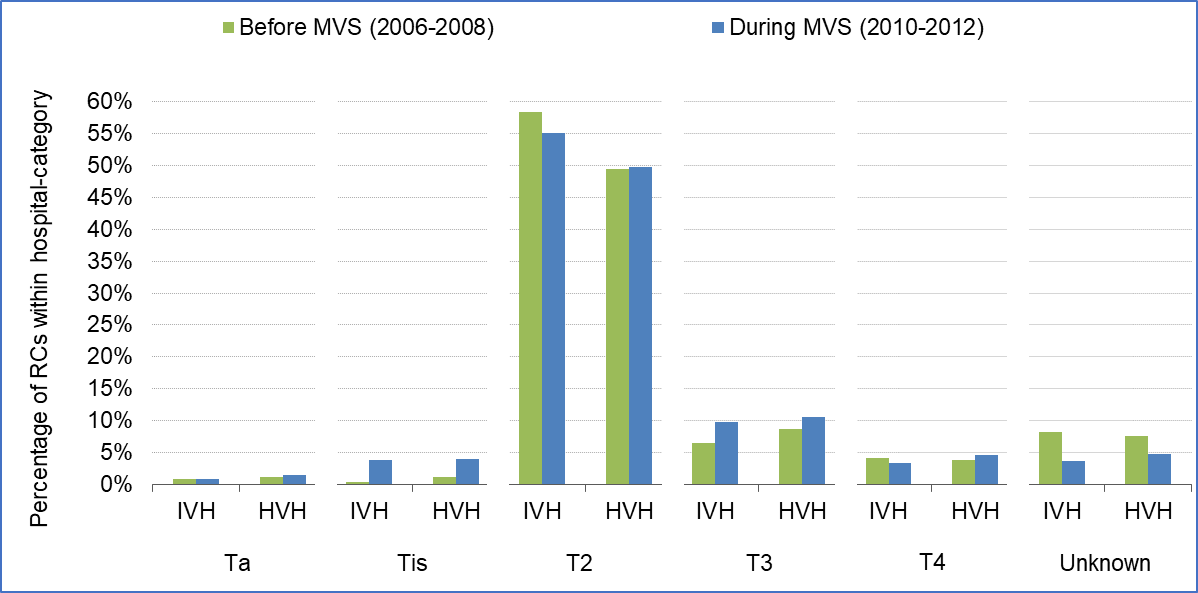


*IVH = intermediate-volume hospitals; HVH= high-volume hospitals; RCs = radical cystectomies; MVS= minimum volume standard;*

Supplementary figure 2: Distribution of other disease stages for intermediate-volume (16-20 RCs/year) and high-volume (≥25RCs/year) hospitals during 2011-2013 (before the introduction of the second minimum volume standard) and 2015-2017 (after the introduction of the second minimum volume standard).


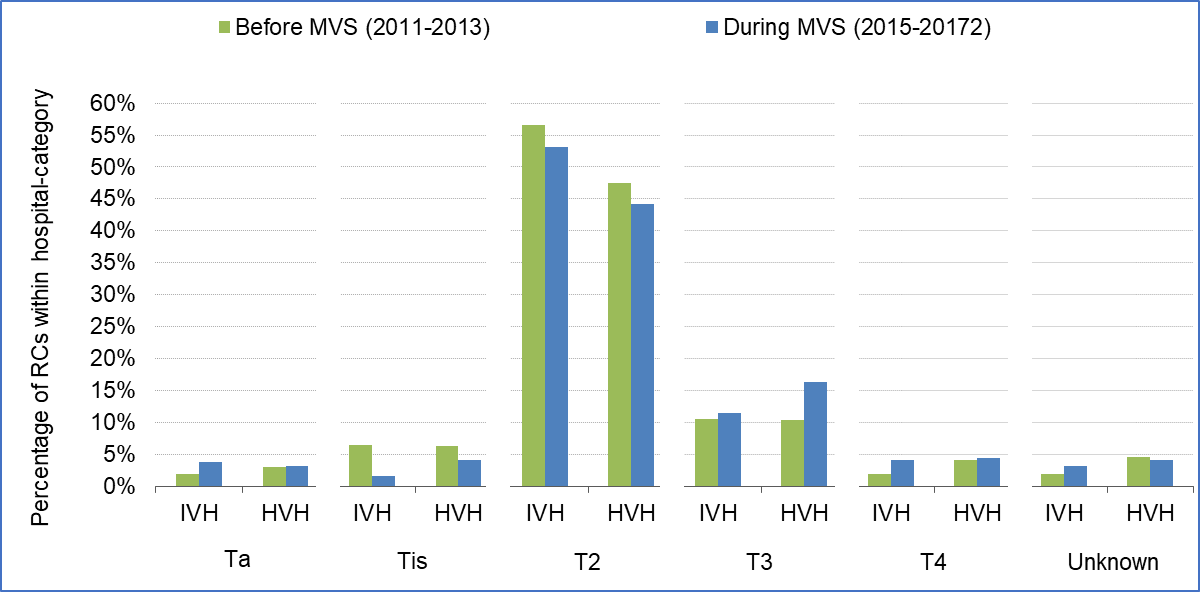


*IVH = intermediate-volume hospitals; HVH= high-volume hospitals; RCs = radical cystectomies; MVS= minimum volume standard;*
